# Simvastatin induces cell cycle arrest and inhibits proliferation of bladder cancer cells via PPARγ signalling pathway

**DOI:** 10.1038/srep35783

**Published:** 2016-10-25

**Authors:** Gang Wang, Rui Cao, Yongzhi Wang, Guofeng Qian, Han C. Dan, Wei Jiang, Lingao Ju, Min Wu, Yu Xiao, Xinghuan Wang

**Affiliations:** 1 Department of Urology, Zhongnan Hospital of Wuhan University, Wuhan, China; 2 Department of Endocrinology, The First Affiliated Hospital of Zhejiang University, Hangzhou, China; 3 Marlene and Stewart Greenebaum Cancer Center, School of Medicine, University of Maryland, Baltimore, MD, USA; 4 Medical Research Institute, School of Medicine, Wuhan University, Wuhan, China; 5 College of Life Science, Wuhan University, Wuhan, China; 6 Center for Medical Science Research, Zhongnan Hospital of Wuhan University, Wuhan, China

## Abstract

Simvastatin is currently one of the most common drugs for old patients with hyperlipidemia, hypercholesterolemia and atherosclerotic diseases by reducing cholesterol level and anti-lipid properties. Importantly, simvastatin has also been reported to have anti-tumor effect, but the underlying mechanism is largely unknown. We collected several human bladder samples and performed microarray. Data analysis suggested bladder cancer (BCa) was significantly associated with fatty acid/lipid metabolism via PPAR signalling pathway. We observed simvastatin did not trigger BCa cell apoptosis, but reduced cell proliferation in a dose- and time-dependent manner, accompanied by PPARγ-activation. Moreover, flow cytometry analysis indicated that simvastatin induced cell cycle arrest at G0/G1 phase, suggested by downregulation of CDK4/6 and Cyclin D1. Furthermore, simvastatin suppressed BCa cell metastasis by inhibiting EMT and affecting AKT/GSK3β. More importantly, we found that the cell cycle arrest at G0/G1 phase and the alterations of CDK4/6 and Cyclin D1 triggered by simvastatin could be recovered by PPARγ-antagonist (GW9662), whereas the treatment of PPARα-antagonist (GW6471) shown no significant effects on the BCa cells. Taken together, our study for the first time revealed that simvastatin inhibited bladder cancer cell proliferation and induced cell cycle arrest at G1/G0 phase via PPARγ signalling pathway.

Bladder cancer (BCa) is one of the most common malignancies of the urinary tract^1^. Approximately 70% BCa patients are non-muscle-invasive disease^2^. BCa has a high risk of recurrence after combined therapy with transurethral resection and intravesical chemotherapy and eventually progressions into muscle-invasive disease with poorer prognosis and higher mortality^3^. For muscle-invasive BCa, the current golden standard treatment is radical cystoprostatectomy^2^, but this therapeutic approach arises many unfavorable outcomes^4^,^5^. Therefore, a more effective strategy for preventing the progression of BCa is urgently needed.

Many risk factors for BCa have been discovered, including aging, smoking, exposure to chemicals, etc.^6^,^7^,^8^. In addition, dietary factors have also been found to contribute to the disease^9^,^10^,^11^. Epidemiologic studies reported that dietary total cholesterol intake and dietary fatty acids intake were associated with elevated risk of several types of cancer, including BCa^12^,^13^. Meanwhile, intracellular cholesterol and fatty acids were important components for cell membrane^14^, especially lipid rafts and cholesterol rich membrane domains, which were required for tumor cell proliferation and metastasis^15^,^16^. Furthermore, intracellular cholesterol biosynthesis was also suggested as an important mechanism for chemotherapy resistance in the BCa cells^17^. Thus, alterations of intracellular lipid metabolism may lead to changes of membrane properties, anti-proliferative, pro-apoptotic and anti-metastasis effects^18^,^19^.

In the current study, our group has profiled several human BCa tissues and normal bladder tissues to generate an novel pathway network^20^, and the bioinformatic analysis promoted us to hypothesize that BCa might be associated with fatty acid and lipid metabolism via Peroxisome Proliferator-Activated Receptor (PPAR) signalling pathway.

The PPARs are a group of nuclear receptors and consist of three distinct subtypes *PPARα*, *PPARβ*/*PPARδ* and *PPAR*γ, which are encoded by distinct genes^21^. PPARs are essential for the regulation of cellular differentiation, development, lipid metabolism and tumorigenesis^22^,^23^. Their activation leads to altered expression of genes involved in cell metabolism, cell growth and stress response. For instance, PPARs increase the transcription level of genes for fatty acid oxidation^24^ and peroxisomal β-oxidation enzymes, which might be important regulators for the homeostasis of the lipid ligands binding to nuclear receptors^25^,^26^. Many reports suggested PPARs modulation in cancer cells by either agonist or antagonist may be a potential treatment for metabolic diseases and cancer including BCa^27^,^28^,^29^. Among those, the PPARγ pathway is particularly critical for the cancer stem cell properties of ErbB2-positive breast cancer cells^30^,^31^, and has been reported to inhibit of ErbB activity in human breast cancer cells^32^.

Statins, 3-hydroxy-3-methyl glutaryl coenzyme A (HMG-CoA) reductase inhibitors, have been well-known for their effects on the rate-limiting step in cholesterol synthesis. Currently, statins are the most common used and effective drugs for old patients with hyperlipidemia, hypercholesterolemia or atherosclerotic diseases^33^,^34^. In addition to their cholesterol reducing effects, increasing evidence from *in vitro* and *in vivo* studies has suggested that statins also have anti-proliferative, pro-apoptotic and anti-metastasis effects in various types of cancer cells^35^,^36^, including BCa cells. However, the exact mechanism is still unknown. Recent studies indicated simvastatin, a broadly used statin drug, could suppress cell proliferation^37^ and induce cell death of breast cancer cells by downregulating ErbB2 via PEA3^38^. In vascular disease, simvastatin has been suggested to inhibit TNFα-induced activation of nuclear factor-kappaB (NFκB) and enhanced expression of *PPARs*^39^. In gallbladder epithelial cells, simvastatin activated the expression of *PPARα/*γ for anti-inflammatory effects through suppression of pro-inflammatory cytokines^40^. Furthermore, by associating with *PPARs*, simvastatin mediated cholesterol and fatty acid reduction^41^. Importantly, simvastatin activated PPARγ-dependent pathway in heart failure^42^ and cardiovascular disease^43^, for example, it attenuated cardiopulmonary-bypass-induced myocardial inflammatory injury by activating PPARγ^44^. Pharmalogical studies suggested combination therapy with PPARs agonist and simvastatin may have important therapeutic significance^45^.

Therefore, we aim to investigate the potential mechanism of the alterations in bladder cancer cells triggered by the treatment of simvastatin.

## Results

### Interaction network analysis for BCa related pathways

**Figure 1:**
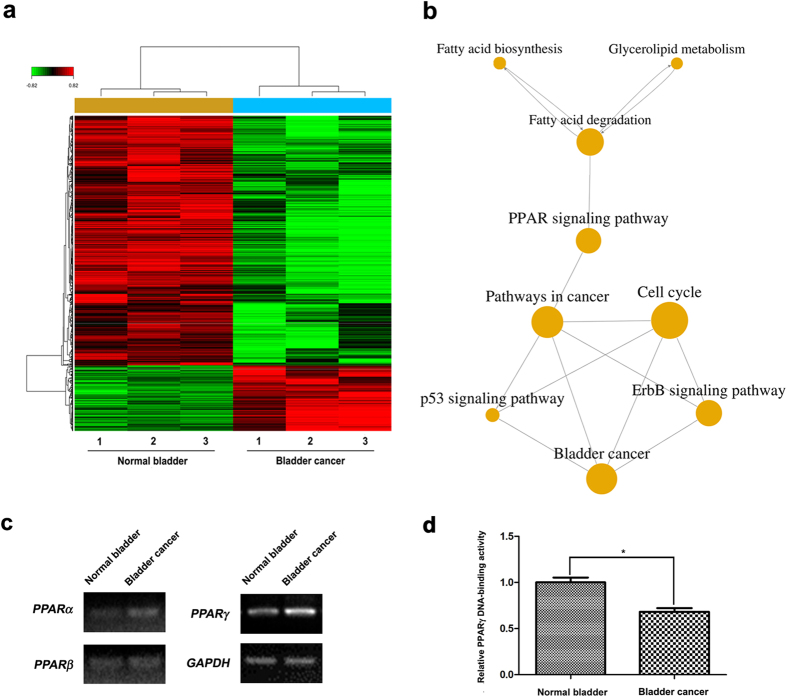
Transcriptome profiling of bladder cancer compared to normal bladder tissues pointed out the PPAR family. (**a**) Heat map of the differentially expressed genes in three bladder cancer tissues compared with three normal bladder tissues. Red color indicated upregulated genes and green color indicated downregulated genes. (**b**) GO-map network analysis by GCBI platform revealed fatty acid biosynthesis and glycerolipid metabolism were linked with bladder cancer via PPAR and ErbB signalling pathways, as well as a close correlation between bladder cancer and cell cycle. (**c**) Semiquantitative RT-PCR analysis for alterations of PPAR family (*PPARα*, *PPARβ* and *PPAR*γ) using pooled total RNA isolated from the three bladder cancer tissues versus three normal bladder tissues. The expression of the *GAPDH* mRNA was used as a loading control. (**d**) ELISA analysis revealed the relative PPARγ DNA-binding activity in the BCa tissues was significantly decreased comparing with the normal bladder tissues (n = 3). *p < 0.05.

The transcriptome differences between human BCa tissues and normal bladder tissues (n = 3, information listed in Table S1) were analysed (Fig. 1a). By using the GCBI platform, significantly altered cell functions were generated (top 20 affected cell functions were listed in Supplementary Fig. S1) and the results indicated mitotic cell cycle (ranked 6), cell proliferation (ranked 13) and positive regulation of cell proliferation (ranked 16) were altered in the BCa. GO and Path-net analysis tools based on KEGG pathway revealed that the cell cycle, pathways in cancer and ErbB signalling pathway were significantly linked to BCa (Fig. 1b). Importantly, fatty acid/lipid metabolism has been suggested to have be close related with bladder cancer via PPAR signalling pathway (Fig. 1b). The genes involved in those significantly altered pathways (cell cycle, p53 signalling pathway, pathways in cancer, PPAR signalling pathway, glycerolipid metabolism, fatty acid degradation, bladder cancer, ErbB signalling pathway, fatty acid biosynthesis) were listed in Supplementary Table S2.

Alteration of PPAR family at the mRNA level was confirmed by semiquantitative RT-PCR analysis, using total RNA isolated from the bladder cancer tissues compared with the normal bladder tissues. Our results shown in Fig. 1c suggested a major inductive expression of *PPAR*γ in the bladder cancer tissues, which is consist with Oncomine database (www.oncomine.org) (Supplementary Fig. S2). However, we did observe a significant reduction of PPARγ DNA-binding activity in the bladder cancer tissues detected by ELISA analysis (Fig. 1d). Taken together, we hypothesized bladder cancer was linked with fatty acid and lipid metabolism via PPARγ signalling pathway.

### Simvastatin inhibited BCa cell viability and proliferation

**Figure 2:**
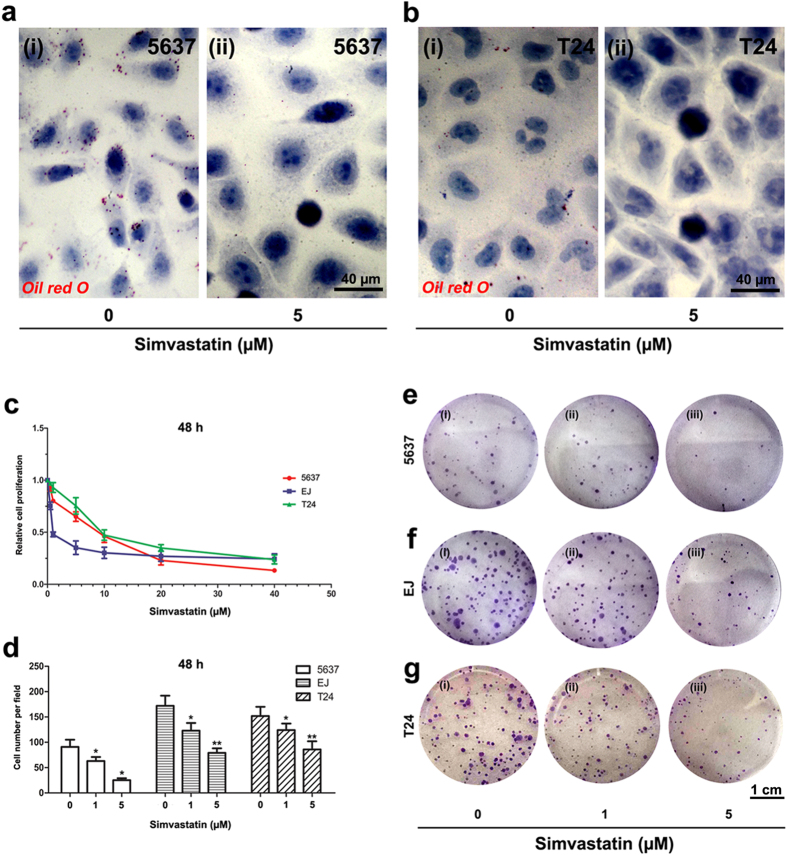
Evaluation of reducing neutral triglycerides and lipids in BCa cells, cell growth, viability and proliferation by simvastatin treatment. (**a,b**) Oil red O staining for simvastatin-treated 5637 (**a**) and T24 (**b**) BCa cells at 0 μM (i) and 5 μM (ii) for 48 h. (**c**) Cell growth and viability were evaluated by MTT assay using distinct BCa cell lines 5637 (red, marked with solid circle), EJ (violet, solid triangle) and T24 (green, solid square) cells treated with 48 h-simvastatin at different concentrations of 0, 0.5, 1, 5, 10, 20 and 40 μM, to determinate the proper concentration of simvastatin treatment on BCa cells. (**e–g**) Cell proliferation of BCa cells 5637 (**e**), EJ (**f**) and T24 (**g**) was evaluated by clonogenic survival assay for the simvastatin-treated BCa cells at 0 (i), 1 (ii) and 5 μM (iii) for 48 h and then cultured in 6-well plates for 15 days. (**d**) Cell number per field in the clonogenic survival assay (cell types and concentration of simvastatin were indicated) was counted and statistically analysed. All values shown were mean ± SD of triplicate measurements and repeated three times with similar results, *p < 0.05, **p < 0.01. The scale bar for (**a,b**) is 40 μm and for (**e–g**) is 1 cm.

Oil red O staining revealed simvastatin could reduce neutral triglycerides and lipids in 5637 (Fig. 2a) and T24 (Fig. 2b) cells after the treatment at 5 μM for 48 h. To further investigate the effect of simvastatin on cell viability in BCa cells (5637, EJ and T24) were treated by simvastatin at different concentrations for 24 h (Supplementary Fig. S3a), 48 h (Fig. 2c), 72 h (Supplementary Fig. S3b). The cell viability determined by MTT assay, suggested a differential effect of simvastatin at 1 and 5 μM after 48 h treatment. Compared to the control group, survival of three BCa cells was considerably inhibited by simvastatin treatment for 48 h in a dose dependent manner (Fig. 2c). Clonogenic survival assay was used to determine the inhibitory effect of simvastatin on cell proliferation (Fig. 2e–g), showing a significant reduction for the colony forming efficiency in the simvastatin-treated BCa cells 5637, EJ and T24, p < 0.05 (Fig. 2d).

### PPARs and ErbB signalling pathways were affected by the treatment of simvastatin

**Figure 3:**
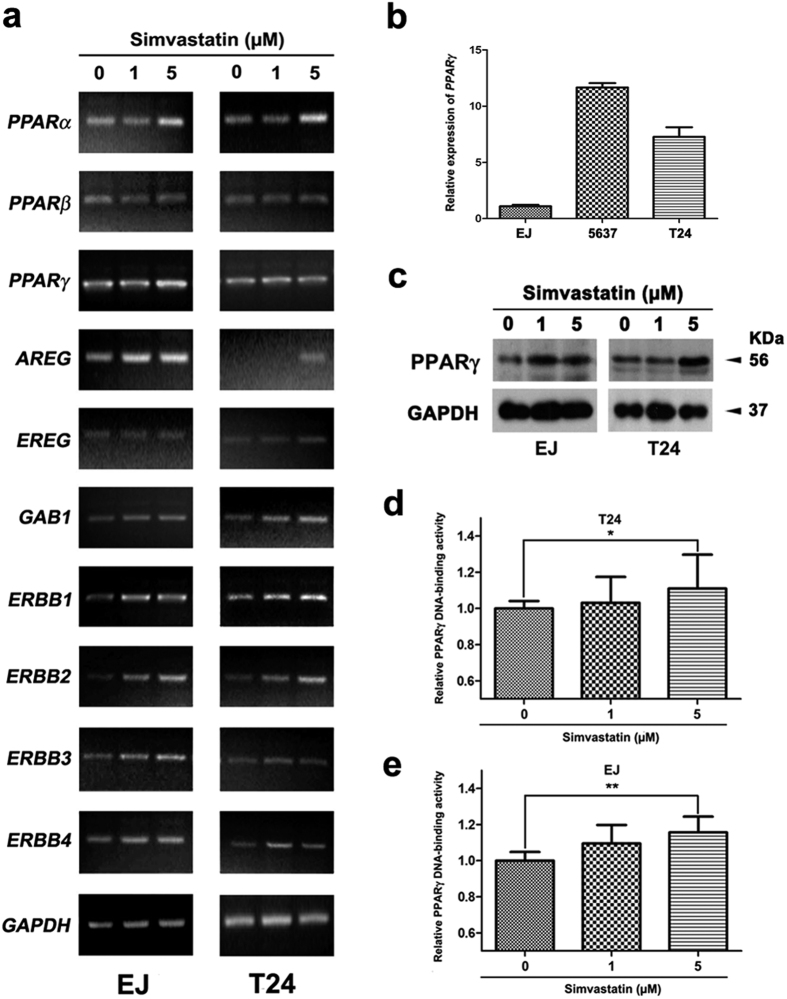
Analysis of genes involved in PPAR and ErbB families. (**a**) Semiquantitative RT-PCR analysis for alterations of PPAR family (*PPARα*, *PPARβ* and *PPAR*γ) and ErbB family (*ERBB1*, *ERBB2*, *ERBB3* and *ERBB4*), as well as ERBB2 related genes (*AREG*, *EREG* and *GAB1*) in EJ and T24 cells after the treatment by simvastatin at 0, 1 and 5 μM for 48 h. The expression level of the *GAPDH* mRNA was used as a loading control. (**b**) qRT-PCR analysis of relative *PPAR*γ mRNA level in the EJ, 5637 and T24 cells. (**c**) Western blot analysis of PPARγ by the simvastatin treatment at 0, 1 and 5 μM. GAPDH was used as a loading control (cell types, concentration of simvastatin treatment and protein masses were indicated). (**d,e**) ELISA analysis revealed the relative PPARγ DNA-binding activity in the T24 (**d**) and EJ (**e**) cells after simvastatin treatment at 0, 1 and 5 μM. All values shown were mean ± SD of triplicate measurements and repeated three times with similar results, *p < 0.05, **p < 0.01.

Semiquantitative RT-PCR analysis revealed upregulation of *PPARα* and *PPAR*γ in the EJ and T24 cells treated with simvastatin (Fig. 3a), similar to the major induction of *ERBB1* and *ERBB2* in the ErbB family (Fig. 3a and Supplementary Fig. S4f). Differentially expressed genes involved in ErbB signalling pathway pointed strong alterations of *GAB1* and *AREG* in the bladder cancer tissues. RT-PCR analysis for the simvastatin-treated BCa cells suggested upregulation of *AREG* and *GAB1*, whereas the effect on *EREG* expression was not strongly altered (Fig. 3a). qRT-PCR analysis revealed the relative expression of *PPAR*γ in the EJ cells was lower than that in 5637 and T24 cells (Fig. 3b), possibly suggesting a more sensitive reaction in the EJ cells by induction of PPARγ. We have observed a strong increase of PPARγ after simvastatin treatment in the BCa cells (EJ and T24) at transcriptional level (Fig. 3a), protein level (Fig. 3c). Importantly, functional analysis using ELISA for DNA-binding activity revealed that PPARγ at functional level was significantly increased in the T24 and EJ cells (Fig. 3d,e).

### Simvastatin suppressed BCa cell migration and invasion

**Figure 4:**
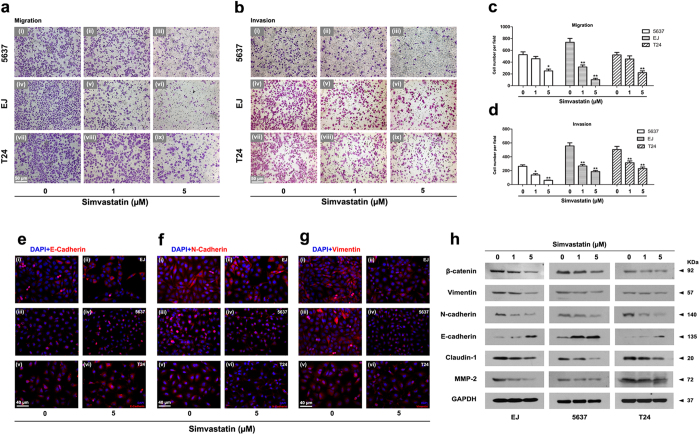
Transwell migration and invasion assay for simvastatin-treated BCa cells. BCa cells 5637 (i–iii), EJ (iv–vi), T24 (vii–ix) were pretreated by simvastatin at 0 (i, iv, vii), 1 (ii, v, viii) and 5 μM (iii, vi, ix) for 48 h. For transwell migration analysis the cells were incubated in the upper transwell chambers for 24 h and the number of migrated cells was counted in five random fields per chamber by phase contrast microscope (**a**) and statistically analysed (**c**). For transwell invasion assay the simvastatin-treated cells were seeded on the transwell chambers precoated with ECM Matrix gel solution, and the number of cells was counted after 48 h incubation using microscope in five random fields per chamber (**b**) and statistically analysed (**d**). All values shown were mean ± SD of triplicate measurements and repeated three times with similar results, *p < 0.05, **p < 0.01. The scale bars for (**a,b**) are 50 μm. (**e–g**) Immunofluorescence staining revealed alterations of E-cadherin, N-cadherin and Vimentin after the simvastatin treatment. The BCa cells, EJ (i, ii), 5637 (iii, iv) and T24 (v, vi), were cultured on the 12 mm coverslips for 24 h, then treated by simvastatin at 0 (i, iii, v) and 5 μM (ii, iv, vi) for 48 h. Immunofluorescence analysis reveals a inducing of E-cadherin (**e**) (red) after the treatment and a reducing of N-cadherin (**f**) (red) and Vimentin (**g**) (red) was noticed by the simvastatin treatment in the BCa cells. Nuclei were stained by DAPI (blue). The images were photographed by fluorescence microscopy. The scale bars for (**e–g**) are 40 μm. (**h**) Western blot analyses of protein (β-catenin, Vimentin, N-cadherin, E-cadherin, Claudin-1 and MMP-2) involved in EMT after 48 h treatment of simvastatin (cell types, concentration of simvastatin treatment and protein masses were indicated). The GAPDH was used as a loading control. 10–30 μg of total protein were loaded per lane.

Cell migration was measured using wound healing (Supplementary Fig. S4a–c) and transwell migration assay (Fig. 4a). After 48 h treatment by simvastatin, migration rates of 5637, EJ and T24 cells were calculated respectively after 12 h (Supplementary Fig. S4d) and 24 h (Supplementary Fig. S4e) incubated by using wound healing assay. The results showed that after 12 h incubation, migration rates for all BCa cells treated by 5 μM simvastatin were significantly reduced (p < 0.05). Interestingly, a sole significant reduction of migrated T24 cells by low concentration of simvastatin treatment (1 μM) was observed (p < 0.01, Supplementary Fig. S4d). After 24 h incubation, migration rates of all BCa cells by simvastatin treatment were significantly decreased (p < 0.05, Supplementary Fig. S4e). Reduction of all BCa migration rates was further confirmed by transwell migration assay (Fig. 4a), suggesting significant decreasing of migration caused by simvastatin treatment at 1 and 5 μM (p < 0.05, Fig. 4c). In addition, invasion of simvastatin-treated BCa cells, suggested by transwell invasion assay (Fig. 4b), was significantly decreased compared to the untreated groups (p < 0.05, Fig. 4d).

### Simvastatin treatment altered the Epithelial-Mesenchymal Transition (EMT)-related protein levels

Proteins involved in the EMT process, including β-catenin, Vimentin, N-cadherin, E-cadherin, Claudin-1 and MMP-2, were analysed by Western blot (Fig. 4h). The study exhibited an upregulation of E-cadherin and downregulation of N-cadherin, β-catenin, Vimentin, Claudin-1 and MMP-2 in the BCa cells after simvastatin treatment. This result was confirmed by immunofluorescence-based analysis as well (Fig. 4e–g), indicating an increase of E-cadherin in EJ, 5637 and T24 cell-cell junctions after simvastatin treatment at 5 μM (Fig. 4e), and a major decrease of N-cadherin in cytoplasm in the BCa cells (Fig. 4f). In addition, Vimentin, a potential bladder cancer marker^46^, was downregulated in cytoplasmic region of all BCa cells after treatment with simvastatin (Fig. 4g).

### Simvastatin treatment induced cell cycle arrest at G0/G1 phase, but exhibited no effect on apoptosis in the BCa cells

**Figure 5:**
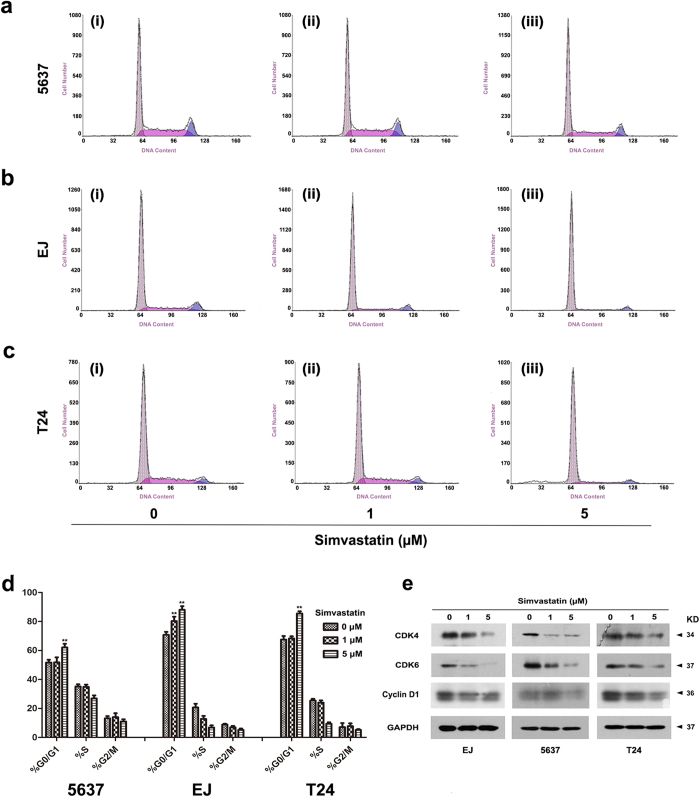
Effect of simvastatin on cell cycle arrest at G0/G1 phase in distinct BCa cells. (**a–c**) Representative flow cytometry analysis for BCa cells 5637 (**a**), EJ (**b**) and T24 (**c**) treated by selected concentration of 0 (i), 1 (ii) and 5 μM (iii) simvastatin for 48 h. (**d**) Alteration of cell cycle was statistically analysed, all values shown were mean ± SD of triplicate measurements and repeated three times with similar results, **p < 0.01. (**e**) Western blot analysis of protein abundance involved in cell cycle (CDK4/6 and Cyclin D1) by using total protein isolated from cell lysates after the simvastatin treatment (cell types, concentration of simvastatin treatment and protein masses were indicated). 10–30 μg of protein were loaded per lane. GAPDH was used as a loading control.

Flow cytometry analysis was performed to reveal the effect of simvastatin on cell cycle (Fig. 5a–c) and apoptosis (Supplementary Fig. S5) in the BCa cells. The study indicated that simvastatin treatment with high concentration (5 μM) for 48 h could increase cell cycle arrest at G0/G1 phase significantly (p < 0.01, Fig. 5d). We also noticed the low concentration treatment of simvastatin (1 μM) could induce the cell cycle arrest significantly in EJ cells as well (p < 0.01), while not in 5637 and T24 cells (Fig. 5d). Proteins levels involved in cell cycle regulation (Cyclin D1 and CDK4/6) were analysed by Western blot (Fig. 5e), revealing a downregulation in a dose-dependent manner after 48 h treatment by simvastatin.

**Figure 6:**
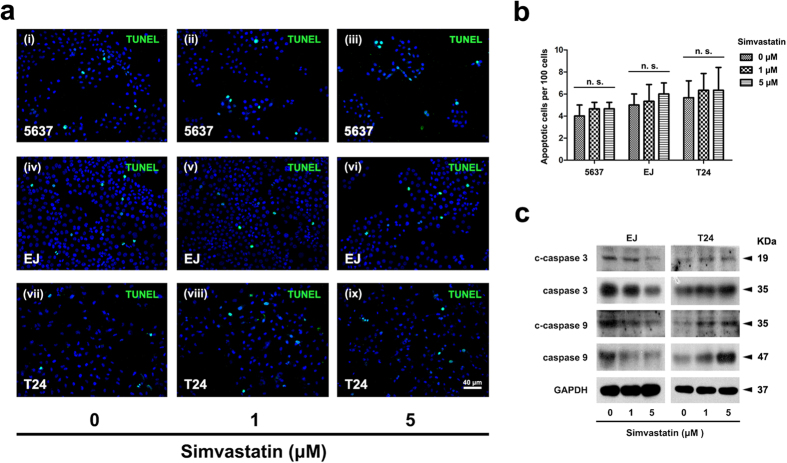
Analysis of BCa cell apoptosis by the treatment of simvastatin. (**a**) TUNEL-test for 5637 (i–iii), EJ (iv–vi) and T24 (vii–ix) after simvastatin treatment at 0, 1 and 5 μM. The scale bar for a is 40 μm. (**b**) Statistical analysis of TUNEL-test revealed no significance (n. s.) of the apoptotic cells per 100 cells in the 5637, EJ and T24 cells after simvastatin treatment at 0, 1 and 5 μM. (**c**) Western blot analysis of cleaved caspase 3 (c-caspase 3), total caspase 3 (t-caspase 3), cleaved caspase 9 (c-caspase 9) and total caspase 9 (t-caspase 9) by using total protein isolated from cell lysates after the simvastatin treatment (cell types, concentration of simvastatin treatment and protein masses were indicated). 10–30 μg of protein were loaded per lane. GAPDH was used as a loading control.

Interestingly, simvastatin could not affect apoptosis in all three tested BCa cell lines, revealed by flow cytometry analysis (Supplementary Fig. S5) and TUNEL test (Fig. 6a,b). Consistently, Western blot analysis suggested the ratios of cleaved caspase 3/9 versus total caspase 3/9 were not considerably changed by simvastatin treatment (Fig. 6c).

### Recovering of BCa cell cycle arrest triggered by simvastatin using PPARγ-antagonist

**Figure 7:**
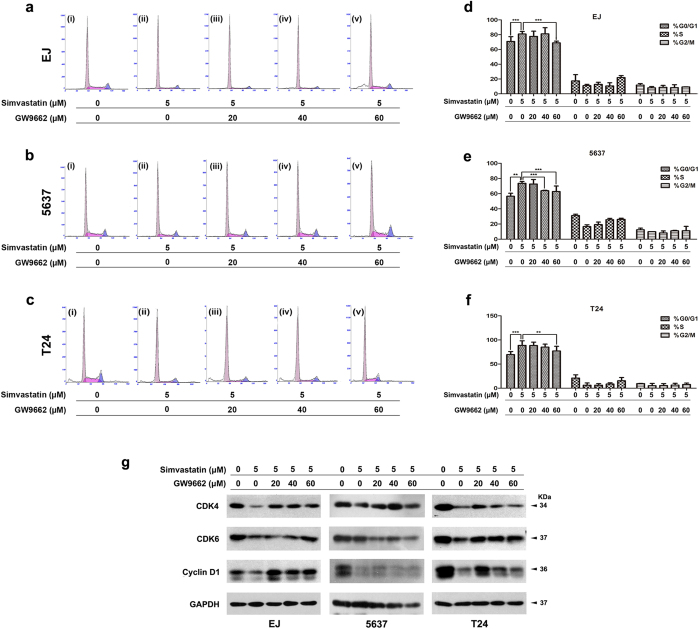
Recovering by PPARγ-antagonist GW9662 for cell cycle arrest at G0/G1 phase triggered by simvastatin in BCa cells. (**a–c**) Representative flow cytometry analysis for the three BCa cells EJ (**a**), 5637 (**b**) and T24 (**c**) treated by GW9662 at 0 μM (i–ii), 20 μM (iii), 40 μM (iv) and 60 μM (v) for 24 h, and continually treated by simvastatin at 0 μM (i) and 5 μM (ii–v) for 48 h. **(d–f**) Recovering of cell cycle arrest at G0/G1 phase by GW9662 were statistically analysed, all values shown were mean ± SD of triplicate measurements and repeated three times with similar results, **p < 0.01, ***p < 0.001. (**g**) Western blot analysis of protein abundance of CDK4/6 and Cyclin D1 using total protein isolated from cell lysates after the simvastatin and GW9662 treatment (cell types, concentration of simvastatin treatment and protein masses were indicated). 10–30 μg of protein were loaded per lane. GAPDH was used as a loading control.

After blocking the PPARγ activity with antagonist GW9662, the effect of simvastatin on BCa cell cycle was studied by flow cytometry analysis (Fig. 7a–c). Similar to Fig. 5a–d, 5 μM simvastatin could trigger cell cycle arrest at G0/G1 phase in the BCa cells EJ (Fig. 7a), 5637 (Fig. 7b) and T24 (Fig. 7c). By incubation with PPARγ-antagonist GW9662 at 20 μM, 40 μM and 60 μM, the cell cycle arrest at G0/G1 phase caused by simvastatin was significantly recovered in a dose-dependent manner of GW9662 (p < 0.01, Fig. 7d–f). Similar to Fig. 5e, we have noticed the reduction of the proteins (CDK4, CDK6 and Cyclin D1) involved in the G0/G1 phase regulation by the sole treatment of simvastatin, however, under GW9662 treatment all the proteins were induced in the BCa cells comparing to the untreated group (Fig. 7g).

## Discussion

Our transcriptome analysis of human bladder cancer tissues and normal bladder tissues suggested that the PPAR signalling pathway could be a link between lipid/fatty acid metabolism and bladder cancer. Simvastatin has been reported to reduce cholesterol and used in hyperlipidemia, hypercholesterolemia and atherosclerotic disease^33^,^34^.

Recent studies revealed an enhanced anti-tumor effect of statins on several human carcinoma cells, including non-small cell lung carcinoma cells, gastric cancer cells, non-melanoma skin cancer cells, breast cancer cells^47^,^48^. The first observation of the effect of simvastatin in bladder cancer cell line suggested a therapeutic advantage combined with doxorubicin-containing chemotherapy in 2015^49^, but the exact mechanism has not been characterized yet. Moreover, simvastatin has been reported to induce breast cancer cell death and deactivate PI3K/AKT and MAPK/ERK signalling pathways^48^. In contrast, our results suggested in the BCa cells simvastatin had no significant effect on apoptosis and cleaved caspase 3/9, but indeed inhibited BCa cell proliferation and trigger cell cycle arrest at G0/G1 phase, by reducing the protein abundances involved in G0/G1 phase regulation (CDK4, CDK6 and Cyclin D1). Importantly, we observed downregulated phosphorylated AKT and p38 in the BCa cells by simvastatin treatment (Supplementary Fig. S4f), suggesting simvastatin might trigger EMT alteration of BCa cells via deactivation of PI3K/AKT and MAPK/ERK signalling pathways, rather than inducing apoptosis.

In addtion, our results revealed an suppressive effect of simvastatin on BCa cell migration and invasion, as well as levels of proteins involved in the EMT. Those effects could mediate important cell functions, dramatically alter cell morphology and loss the cell-to-cell tight junction. Accumulating evidences suggested EMT was involved in cancer invasion and metastasis^50^,^51^. The association of Cytokeratin and Vimentin was also reported in the genesis of transitional cell carcinoma of urinary bladder patients, and therefore could be a helpful marker in the early diagnosis of transitional bladder carcinoma^46^. In our study, an upregulation of epithelial marker E-cadherin protein level was noticed in the simvastatin-treated BCa cells (Fig. 4h). Consistently, the potential bladder cancer marker - Vimentin was downregulated in cytoplasmic region of all BCa cells treated with simvastatin (Fig. 4g).

To better understand the effects of simvastatin on bladder cancer, we analysed the alterations of PPAR family indicated by our microarray result in detail. RT-PCR analysis indicated an increase of *PPAR*γ mRNA level in the bladder cancer tissue. However, functional assay revealed a reduced DNA-binding activity of PPARγ protein in the BCa tissues. Furthermore, upon the treatment with simvastatin, the BCa cells showed an increase of PPARγ at transcriptional (Fig. 3a), protein (Fig. 3c) and functional levels (Fig. 3d,e), which was similar to the result from hepatocarcinoma HepG2 cells^52^. As previously reported, PPARγ could be activated by simvastatin-induced dysregulation of cholesterol synthesis in adult human glial progenitors^44^ and inflammatory response in rat cardiopulmonary bypass^53^. However, the detailed regulating mechanism remains not clarified.

Furthermore, our results suggested the increased *PPAR*γ could not trigger BCa cell apoptosis (Fig. 6), but with a time- and concentration-dependent manner to inhibit cell proliferation and increasing number of cells at the G0/G1 phase (Fig. 5). Since our transcriptome analysis suggested the link between lipid/fatty acid metabolism and PPAR signalling pathway in bladder cancer, and we noted an induction of PPARγ at protein and functional level in the simvastatin-treated BCa cells. Therefore, we treated the BCa cells with PPARγ-antagonist GW9662 to suppress the activity of PPARγ. We did not see a recovered phenotype of the simvastatin-induced inhibition of the migration rate (Supplementary Fig. S7), but we noticed the recovered effect on cell cycle arrest at G0/G1 phase caused by simvastatin in all three BCa cell lines. This was further supported by our observed increased protein level (CDK4, CDK6 and Cyclin D1) involved in the G0/G1 phase in the GW9662-treated BCa cells, especially in the EJ and T24 cells. We also noticed that treatment with PPARα-antagonist GW6471 exhibited no significant effects of recovering the cell cycle arrest triggered by simvastatin on the three BCa cells (Supplementary Fig. S6). This might indicate simvastatin could induce BCa cell cycle arrest at G0/G1 phase mainly through PPARγ, rather than PPARα.

In our study, we observed a strong upregulation of *ERBB1–2* mRNA level (Fig. 3a) and a downregulation of ERBB1 protein (Supplementary Fig. S4f) upon treated with simvastatin in the BCa cells. We also noticed alterations of *GAB1, AREG* and *EREG* mRNA levels (Fig. 3a) by simvastatin treatment in the BCa cells. These two together suggested a potential link between ERBB signalling pathway and tumor progression, which has been indicated in previous report^54^,^55^, however, further studies are needed to clarify the underlying mechanism between ERBB signalling pathway and tumorigenesis of bladder cancer.

In conclusion, our study suggested that simvastatin could inhibit proliferation and EMT and trigger cell cycle arrest at G0/G1 phase via the PPARγ signalling pathway in bladder cancer cells.

## Materials and Methods

### Human bladder tissue samples

Three bladder cancer (stage II) tissue samples were collected from patients after surgery by radical resection, and three normal bladder tissue samples were collected from donors by accidental death in Zhongnan Hospital of Wuhan University. Histological diagnosis of the human bladder tissues were examined by two pathologists independently. Samples were obtained from operation room, snap-frozen immediately and stored in liquid nitrogen. Informed consent was obtained from all subjects. Information of the patients and donors (without personal information) was listed in Supplementary Table S1. This study was approved by the Ethics Committee at Zhongnan Hospital of Wuhan University (approval number: 2015029). The sample collection and treatment for total RNA isolation were carried out in accordance with the approved guidelines.

### BCa cell lines

Human BCa cell lines T24 (transitional cell carcinoma, Cat. #SCSP-536) and 5637 (grade II carcinoma, Cat. #TCHu 1) were kindly provided by Stem Cell Bank, Chinese Academy of Sciences in Shanghai. EJ cells (carcinoma, Cat. #CL-0274) was purchased from Procell Co. Ltd., Wuhan, China. The BCa cell lines were identified by the China Centre for Type Culture Collection in Wuhan, China. Cells were cultured in RPMI-1640 medium (Gibco, China) with 1% penicillin G sodium/streptomycin sulphate and 10% fetal bovine serum (FBS) (Gibco, Australia) in a humidified atmosphere consisting of 95% air and 5% CO_2_ at 37 °C.

### Drug treatment for BCa cells

#### MTT test for simvastatin treatment

BCa cells were seeded in 96-well plates (3,000 cells per 100 μl medium), cultured for 24 h and treated by simvastatin (0, 0.5, 1, 5, 10, 20 and 40 μM, diluted in DMSO) for 24 h (Fig. S3a), 48 h (Fig. 2c) and 72 h (Fig. S3b). After adding 10 μl MTT (5 mg/ml) to each well and incubating for 4 h at 37 °C, removing the medium and dissolving formazan precipitate in 100 μl DMSO, absorbance at 490 nm was measured by Rayto-6000 system (Rayto, China). MTT test were exhibited as “Relative cell proliferation”. The absorbance value of each measurement was normalized to the value of simvastatin at 0 μM (DMSO control) for each cell type, calculated as: “Relative cell proliferation” = “MTT Absorbance value of simvastatin-treated cells”/“MTT Absorbance value of simvastatin-untreated cells”. All the following experiments using simvastatin-treated BCa cells were generated as: BCa cells were cultured for 24 h and treated by simvastatin 0 μM (DMSO control), 1 μM and 5 μM for 48 h.

#### PPARs-antagonist treatment

After culture for 24 h, BCa cells were treated by PPARα-antagonist GW6471 (0, 10 and 20 μM) (Sigma-Aldrich, Cat. #G5045) and PPARγ-antagonist GW9662 (0, 20, 40 and 60 μM) (Sigma-Aldrich, Cat. #M6191) for 24 h. The PPARs-antagonists treated BCa were subsequently subjected to simvastatin treatment for 48 h.

### RNA expression analyses

#### Total RNA isolation from bladder cells and tissues

Total RNA was isolated from BCa cells and bladder tissues by RNeasy Mini Kit from Qiagen (Cat. #74101), combined with QIAshredder (Qiagen, Cat. #79654) using a centrifuge (Eppendorf, Cat. #5424), according to the manufacturer’s protocol. Each RNA preparation was subjected to DNase I digestion (Qiagen, Cat. #79254) to remove genomic DNA. Quantity of isolated RNA was assessed with a NanoDrop^®^ ND-2000 UV-Vis spectrophotometer (Thermo Scientific, USA).

#### Microarray analysis of mRNA alterations from bladder tissues

Three human BCa and three normal bladder tissues were obtained for RNA extraction and hybridization. Biotinylated cDNA were prepared according to the standard Affymetrix protocol from 250 ng total RNA using Ambion^®^ WT Expression Kit. Following labelling, 5.5 μg of cDNA were hybridized for 16 h at 45 °C on GeneChip Human Transcriptome Array 2.0 in Hybridization Oven 645. GeneChips were washed and stained in the Affymetrix Fluidics Station 450, scanned by Affymetrix^®^ GeneChip Command Console (AGCC) installed in GeneChip^®^ Scanner 3000 (7G). Data were analysed with Robust Multichip Analysis (RMA) algorithm using Affymetrix default analysis settings and global scaling as normalization method. Bladder cancer related genes and pathways were analysed by Gene ontology (GO) and Pathway-Relation-Network (Path-net) analysis tools based on Kyoto Encyclopedia of Genes and Genomes (KEGG) Pathway Database using Gene Cloud of Biotechnology Information (GCBI Platform, Shanghai, China) (www.gcbi.com.cn)^56^. The microarray data was submitted to the Gene Expression Omnibus (GEO) database (accession number: GSE76211). All data are MIAME compliant.

### Reverse transcription and semiquantitative RT-PCR

First-strand cDNA was synthesized from 1 μg of total RNA using RevertAid First Strand cDNA Synthesis Kit (Thermo Scientific, China). For the polymerase chain reactions (PCR) 400 ng cDNA per 25 μl reaction was used. All primers were tested for optimizing annealing temperatures and conditions using gradient PCRs (Bio-Rad iCycler, Cat. #T100). RT-PCR primers were listed in Supplementary Table S3.

### Quantitative real time PCR (qRT-PCR)

400 or 500 ng cDNA were used for each reaction of the PCR in a final volume of 25 ml. All primers conducted with the SYBR Premix Ex Taq II (Takara Bio, China) were tested for optimal annealing temperatures and PCR conditions were optimized with gradient PCRs on a Bio-Rad iCycler (Cat. #CFX96). Primer sequences and annealing temperatures are summarized in Supplementary Table S3. Values were normalized for amplified β-actin alleles.

### Protein Analyses

#### Isolation of total protein

Simvastatin-treated BCa cells were sonicated and lysed in RIPA buffer containing protease inhibitor and phosphatase inhibitor (Sigma-Aldrich, USA) on ice for 30 min. Cell lysates were centrifuged at 12,000 g for 15 min to collect supernatant. Protein concentration was determined by Bradford protein assay (Bio-Rad, Germany) using Bovine serum albumin (BSA) as standard.

#### Western blots

Briefly, total protein was separated using 7.5–12.5% SDS-PAGE and transferred to PVDF membrane (Millipore, USA). Membranes were blocked in 5% fat-free milk and incubated with primary antibodies (Supplementary Table S4) for overnight at 4 °C. After washing, membranes were incubated with secondary antibody (Supplementary Table S5) for 2 h at room temperature (RT). Bands were detected using an enhanced chemiluminescence kit (Bio-Rad, USA) and blots were exposured to Kodak Biomax MR Films.

#### Enzyme linked immunosorbent assay (ELISA)

PPARγ DNA-binding activity was assessed using the PPARγ transcription factor assay kit (Abcam, USA) according to the manufacturer’s protocol. Briefly, preparation of the nuclear fraction from the BCa tissues and normal bladder tissues (n = 3, biological replicates) using the Nuclear and Cytoplasmic Extraction Reagents (Thermo scientific, USA). Then, 10 μl of the nuclear extract was added to the provided wells precoated with a specific double-stranded DNA (dsDNA) sequence containing the peroxisome proliferator response element. A primary anti-PPARγ antibody was then added, followed by the HRP-conjugated secondary antibody. The absorbance was read at 450 nm by a microplate reader (SpectraMax M2, Molecular Devices, USA) after addition of developing and stop solution, and statistically analysed by t-test. For ELISA analysis of PPARγ in BCa cells (T24 and EJ) by the treatment of simvastatin (0, 1 and 5 μM), three technical replicates for each group were applied and conducted using the same procedure above, and statistically analysed by t-test.

#### Immunofluorescence staining

BCa cells were seeded on the 12 mm coverslips after simvastatin treatment and washed with ice cold phosphate-buffered saline (PBS, pH 7.4), fixed with 4% paraformaldehyde (PFA) for 30 min, treated by 0.1% Triton X-100 and blocked in goat serum for 30 min at room temperature, incubating with primary antibody (Supplementary Table S4) for 2 h at room temperature, washing with PBS and incubating with Cy3-labelled or FITC- labelled secondary antibody (Supplementary Table S4) for 1 h. Nuclei were labelled with DAPI (2 μg/ml). Immunofluorescence staining were analysed using a fluorescence microscope (Olympus, Cat. #IX73).

### Cell culture analysis

#### Lipid analysis using Oil red O (ORO) staining

Simvastatin-treated BCa cells on 12 mm coverslips were fixed by ice cold 10% formalin for 5 min, rinsed in distilled water and 60% isopropanol (Sigma-Aldrich, USA). Coverslips were stained with the freshly prepared ORO (Sigma-Aldrich, USA) working solution (30 ml ORO stock solution and 20 ml distilled water) for 15 min at room temperature and rinsed with 60% isopropanol, mounted in glycerol gelatin medium (Sigma-Aldrich, USA) and inspected with a phase contrast microscope (Leica, Cat. #DMI 1).

#### Clonogenic survival assay

After simvastatin treatment, BCa cells were seeded in 6-well plates (800 cells per well) and grew into colonies for approximately 15 days. Colonies were emerged and fixed by 4% PFA for 30 min, then stained by crystal violet, counted and photographed.

#### Flow cytometry analysis for cell cycle arrest

Simvastatin-treated BCa cells were harvested and washed with cold PBS twice. 1 × 10^6^ cells were harvested and fixed in 70% ice cold ethanol (−20 °C, overnight), washing again with cold PBS and incubating with RnaseA (20 μg/ml in PBS), stained by propidium iodide (50 μg/ml) for 30 min (Sigma-Aldrich, USA) at room temperature in the dark. Cell cycle were assessed on a flow cytometry (Beckman, Cat. #FC500).

#### Flow cytometry assay for apoptosis

Apoptosis was assessed by annexin V-fluorescence isothiocyanate (FITC)/PI apoptosis detection kit (BD biosciences, USA) according to the manufacturer’s instructions. Simvastatin-treated BCa cells were harvested, washed twice with cold PBS and centrifuged at 200 g for 10 min, resuspended in 100 ml 1x binding buffer, incubating with FITC- annexin V and PI for 15 min at room temperature in the dark. 400 μl of 1x binding buffer was added to each tube and assessed on flow cytometry.

#### TUNEL assay

After simvastatin treatment, the BCa cells on 12 mm coverslips were fixed by 4% PFA for 25 min at RT and washed three times by PBS. The cells were continuously incubated with 0.1% Triton X-100 for 2 min and washed by PBS. Apoptosis was detected with a TUNEL assay (Roche Applied Science, Germany), according to the manufacturer’s instructions. Nuclei were labelled with DAPI (2 μg/ml). Immunofluorescence staining were analysed by fluorescence microscope.

#### Wound healing assay

Simvastatin-treated BCa cells were seed in 6-well plates and scratched, washed with PBS, adding 0.5% FBS medium to allow cells moving into the gap, photographed by phase contrast microscope at 0 h, 12 h and 24 h in several pre-marked spots. Migration rate was calculated as the proportion of initial scratch distant of each sample and the mean distance between both borderline remaining cell free after migration.

#### Transwell chamber migration and invasion assay

For migration assay, BCa cells were treated with simvastatin and seeded (6 × 10^4^ cells per chamber) in a serum-free medium in the upper transwell chambers (Corning, USA) and medium containing 10% FBS in the lower chambers to induce cell migration. After 24 h, the cells were fixed with 4% PFA and stained by 0.1% crystal violet, migrated cell number was counted by phase contrast microscope and statistically analysed. To perform the invasion assay, transwell chambers were precoated with ECM Matrix gel solution (Sigma-Aldrich, USA) to grow the simvastatin-treated cells (8 × 10^4^ cells per chamber) for 24 h. Residual cells on the upper transwell chambers were counted and statistically analysed in five random fields per chamber.

### Statistical analyses

All analyses were performed three times and represent data from three individual experiments. Two-tailed Student’s t-test and one-way analysis of variance (ANOVA) were used to evaluate statistical significance of differences of data. Statistical analyses were performed with SPSS 16.0. Statistical significance was set at probability values of p < 0.05.

## Additional Information

**How to cite this article**: Wang, G. *et al*. Simvastatin induces cell cycle arrest and inhibits proliferation of bladder cancer cells via PPARγ signalling pathway. *Sci. Rep.* **6**, 35783; doi: 10.1038/srep35783 (2016).

## Supplementary Material

Supplementary Information
