# Supplementary material for: Simvastatin induces cell cycle arrest and inhibits proliferation of bladder cancer cells via PPARγ signalling pathway
Source: Sci Rep. 2016 Oct 25;6:35783. doi: 10.1038/srep35783 (PMC5078845; doi:10.1038/srep35783)
Supplement: Supplementary Information [file srep35783-s1.pdf]

The title: **Simvastatin induces cell cycle arrest and inhibits proliferation of bladder cancer cells via PPARγ signalling pathway**

The authors: Gang Wang, Rui Cao, Yongzhi Wang, Guofeng Qian, Han C. Dan, Wei Jiang, Lingao Ju, Min Wu, Yu Xiao, Xinghuan Wang

Supplementary Information 1

Supplementary Figure S1. Top 20 ranking of affected cell function by mRNA microarray using bladder cancer tissue, compared with normal bladder tissue. Based on GO analysis from GCBI platform, the alteration of mitotic cell cycle (ranked 6), cell proliferation (ranked 13) and positive regulation of cell proliferation (ranked 16) in the human bladder cancer were noticed.

Supplementary Figure S2. Upregulation of *PPARγ* mRNA expression in bladder cancer analysed by Oncomine database. Analysis using the Oncomine database (www.oncomine.org) revealed an increased *PPARγ* at mRNA level in bladder cancer versus normal bladder tissues.

Supplementary Figure S3. Evaluation of BCa cell growth and viability by simvastatin treatment for 24 and 72 h. Cell growth and viability were analysed by MTT assay using 5637 (red, marked with solid circle), EJ (violet, solid triangle) and T24 cells (green, solid square) treated by simvastatin at different concentrations of 0,

0.5, 1, 5, 10, 20 and 40 μM, cultured for 24 (**a**) and 72 h (**b**), to determine the correct concentration of simvastatin treatment on BCa cells.

Supplementary Figure S4. Analysis of BCa cell migration and protein abundance of the related pathways after simvastatin treatment by wound healing assay. Three BCa cells 5637 (**a**), EJ (**b**) and T24 (**c**) in 6-well plates were divided into three groups for treatment by 0 (i-iii), 1 (iv-vi) and 5 μM (vii-ix) simvastatin after the wound was generated. Wound healing assay was monitored by phase contrast microscope at 0 (i, iv, vii), 12 (ii, v, viii) and 24 h (iii, vi, ix). (**d**) and (**e**) revealed statistical analysis for cell migration after simvastatin treatment at 12 h and 24 h respectively. * $p<0.05$, ** $p<0.01$. (**f**) Western blot analysis of phosphorylated AKT (p-AKT), total AKT (t-AKT), phosphorylated GSK3β (p-GSK3β), total GSK3β (t-GSK3β), ERBB1, phosphorylated p38 (p-p38) and total p38 (t-p38) in the EJ and T24 cells by simvastatin treatment at 0, 1 and 5 μM. Protein abundance of GAPDH was used as a loading control.

Supplementary Figure S5. Flow cytometry analysis for BCa cell apoptosis by FITC-Annexin V/PI staining assay after simvastatin treatment. Three types of BCa cells, 5637 (**a**), EJ (**b**) and T24 (**c**), were treated by simvastatin at 0 (i), 1 (ii) and 5 μM (iii) for 48 h. Cell apoptosis was using flow cytometry analysis (representatively indicated in **a-c**), revealing no considerably differences of apoptosis by the effect of simvastatin.

Supplementary Figure S6. Effect of PPARα-antagonist GW6471 for cell cycle alterations triggered by simvastatin in BCa cells. The three BCa cells EJ (**a**), 5637 (**b**) and T24 (**c**) were treated by GW6471 at 0 μM (i-ii), 20 μM (iii), 40 μM (iv) and 60 μM (v) for 24 h, and continually treated by simvastatin at 0 μM (i) and 5 μM (ii-v) for 48 h. Alterations of cell cycle were measured by flow cytometry analysis (representatively indicated in **a-c**) and statistically analysed (**d-f**), * $p<0.05$, ** $p<0.01$, *** $p<0.001$.

Supplementary Figure S7. Effect of PPARγ-antagonist GW9662 on migration of the simvastatin-treated BCa cells. The three BCa cells 5637 (**a**), EJ (**b**) and T24 (**c**) were treated by GW9662 at 0 μM (i-ii), 20 μM (iii) and 40 μM (iv) for 24 h, and continually treated by simvastatin at 0 μM (i) and 5 μM (ii-v) for 48 h. Migration and invasion were revealed by the transwell assay, suggesting no strong effect of GW9662 on recovering the reduced migration and invasion rate of the BCa cells by simvastatin treatment. The scale bars for a-c are 50 μm.

Supplementary Figure S1. Top 20 ranking of affected cell function by mRNA microarray using bladder cancer tissue, compared with normal bladder tissue.

Function Analysis

543 in total

序号	GO ID	GO Name	Diff Gene Counts in GO	Gene Amount
1	GO:0007165	signal transduction	111	1030
2	GO:0007596	blood coagulation	73	465
3	GO:0006955	immune response	58	351
4	GO:0051301	cell division	53	295
5	GO:0007155	cell adhesion	63	454
6	GO:0000278	mitotic cell cycle	53	363
7	GO:0000236	mitotic prometaphase	28	100
8	GO:0044281	small molecule metabolic process	104	1363
9	GO:0030198	extracellular matrix organization	37	210
10	GO:0045087	innate immune response	60	554
11	GO:0006954	inflammatory response	43	295
12	GO:0000087	M phase of mitotic cell cycle	35	198
13	GO:0008283	cell proliferation	45	336
14	GO:0030168	platelet activation	35	204
15	GO:0007067	mitosis	32	182
16	GO:0008284	positive regulation of cell proliferation	48	411
17	GO:0045944	positive regulation of transcription from RNA polymeras…	63	708
18	GO:0006915	apoptotic process	60	654
19	GO:0006936	muscle contraction	22	95
20	GO:0035556	intracellular signal transduction	36	284

Supplementary Figure S2. Upregulation of *PPARγ* mRNA expression in bladder cancer analysed by Oncomine database.

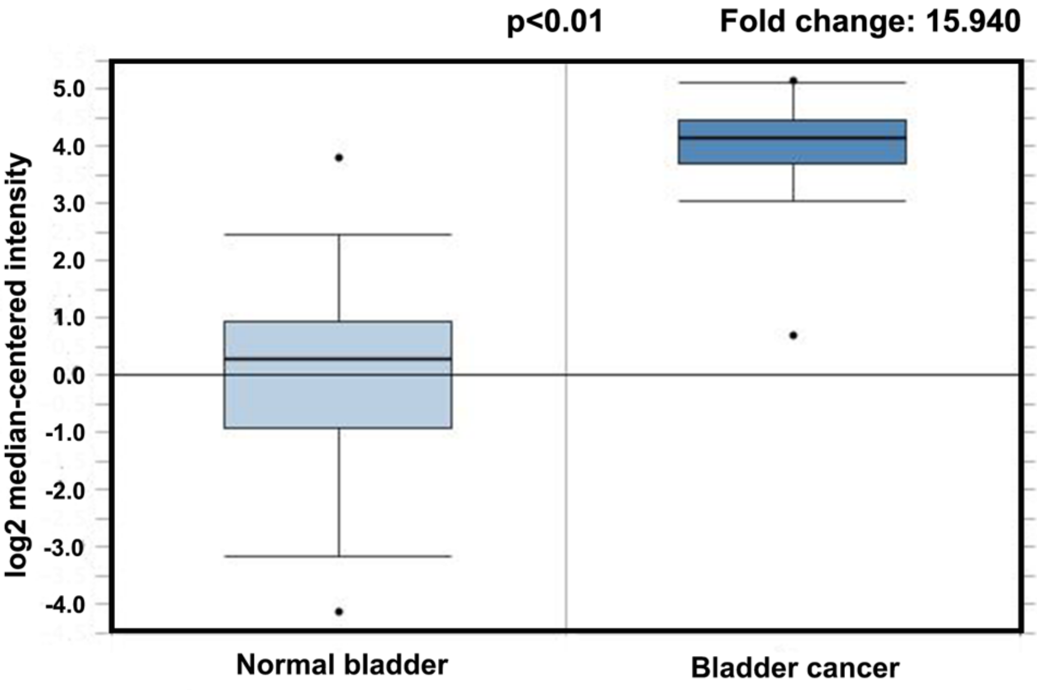

Supplementary Figure S3. Evaluation of BCa cell growth and viability using MTT test after simvastatin treatment for 24 and 72 h.

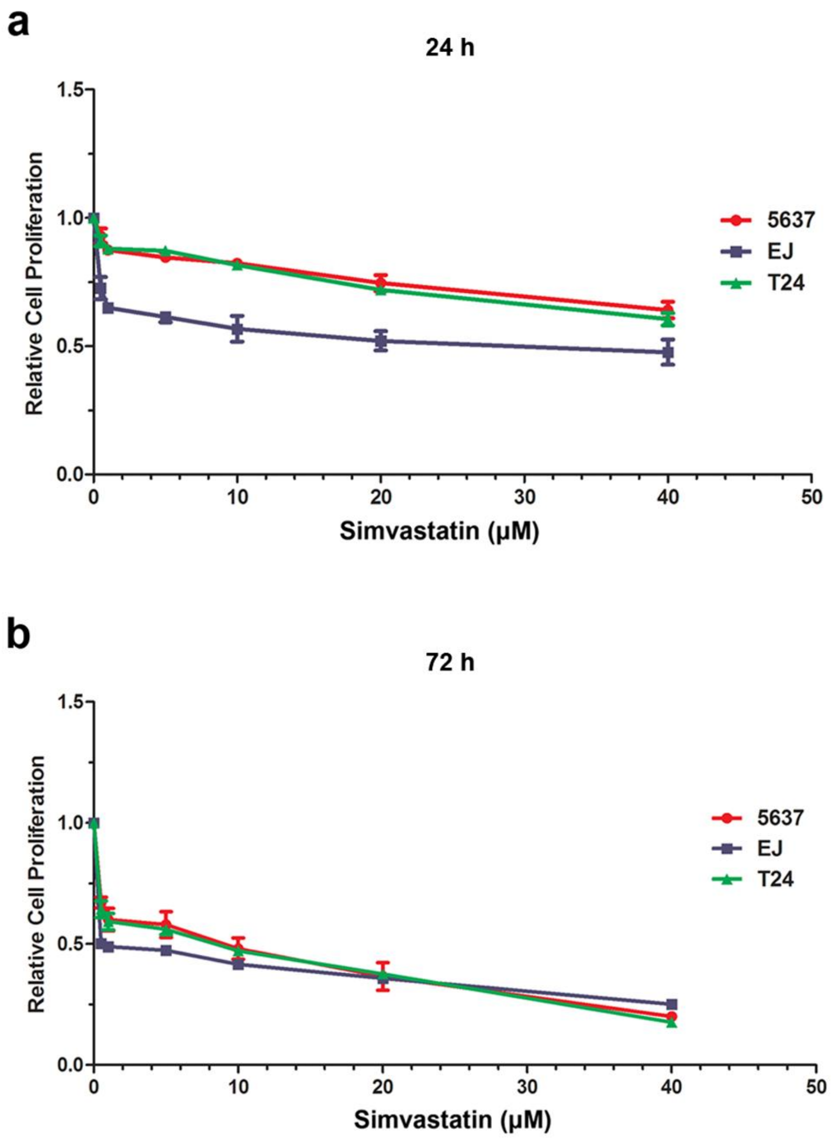

Supplementary Figure S4. Analysis of BCa cell migration and protein abundance of the related pathways after simvastatin treatment by wound healing assay.

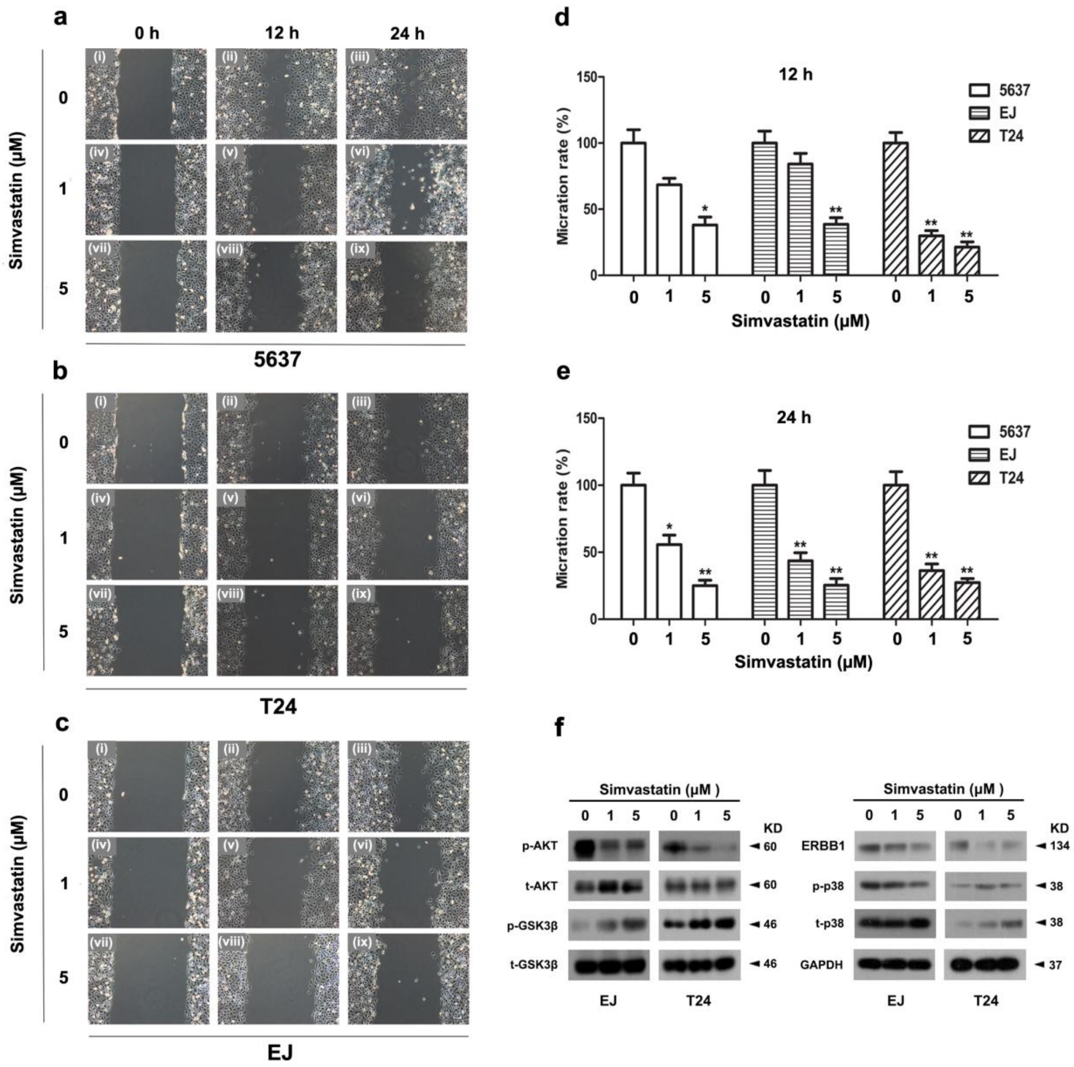

Supplementary Figure S5. Representative flow cytometry analysis for BCa cell apoptosis by FITC-Annexin V/PI staining assay after simvastatin treatment.

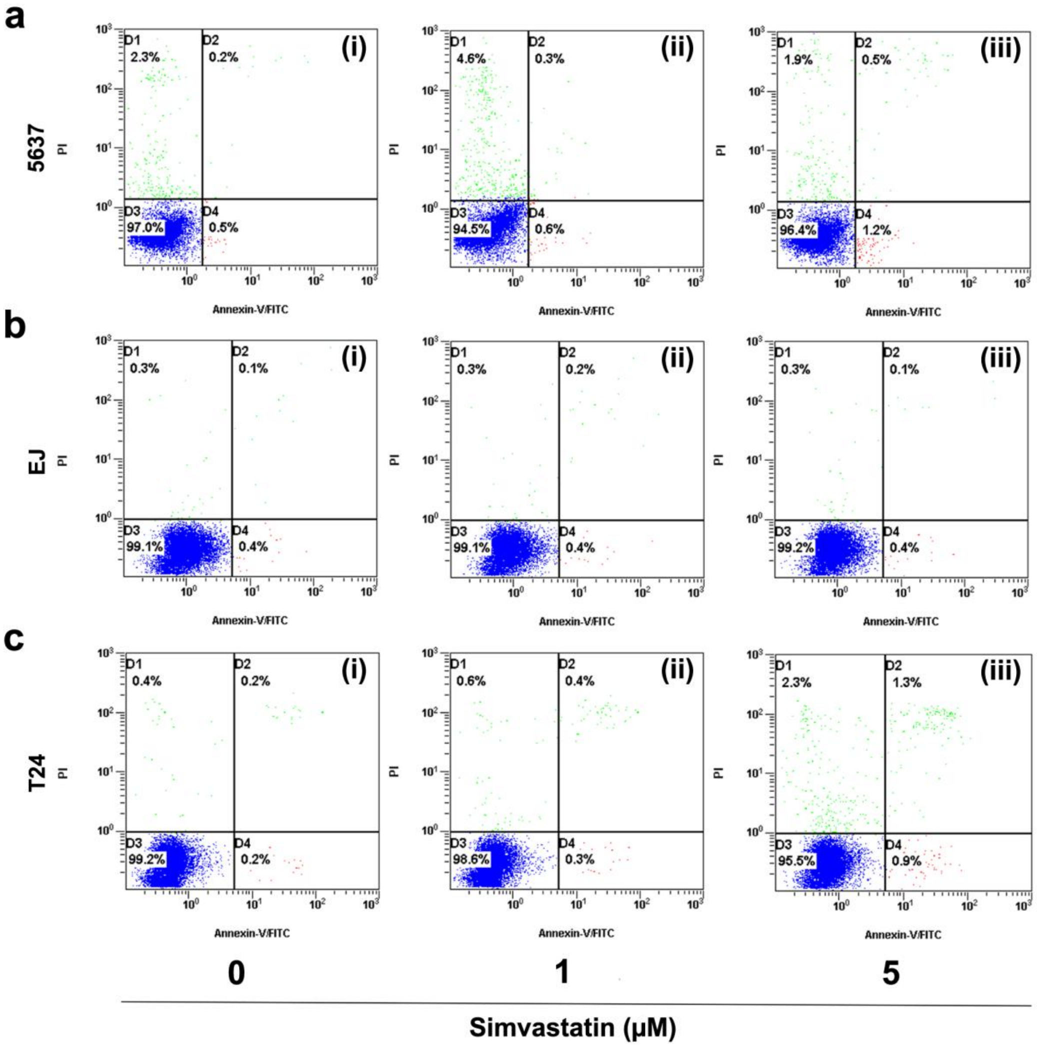

Supplementary Figure S6. Effect of PPARα-antagonist GW6471 for cell cycle alterations triggered by simvastatin in BCa cells.

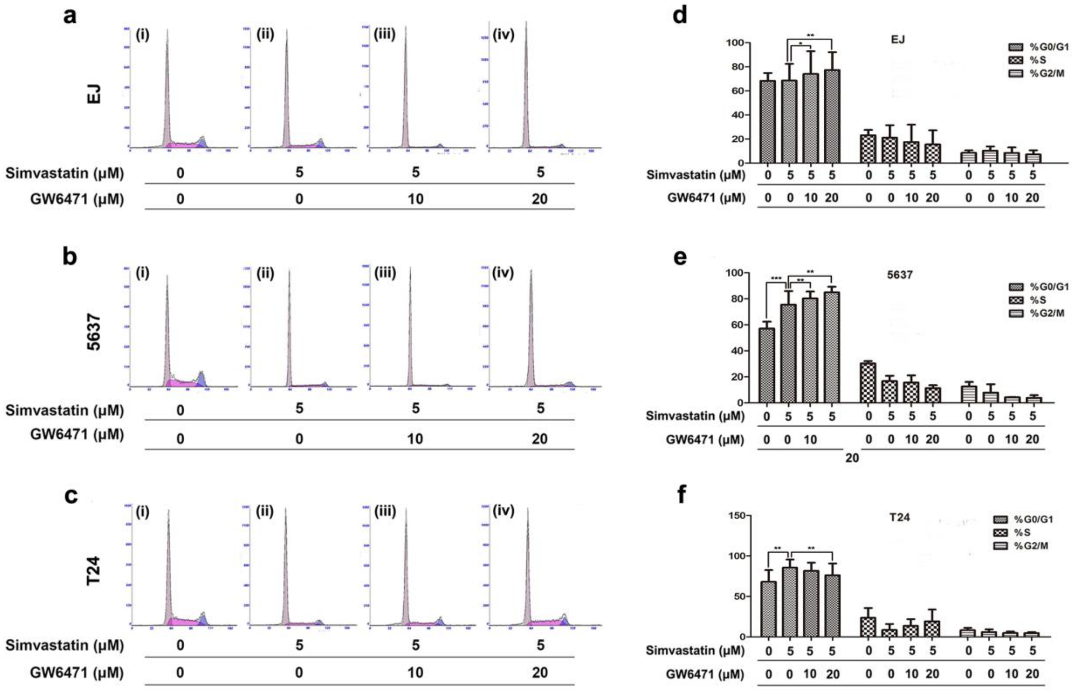

Supplementary Figure S7. Effect of PPARγ-antagonist GW9662 on migration of the simvastatin-treated BCa cells.

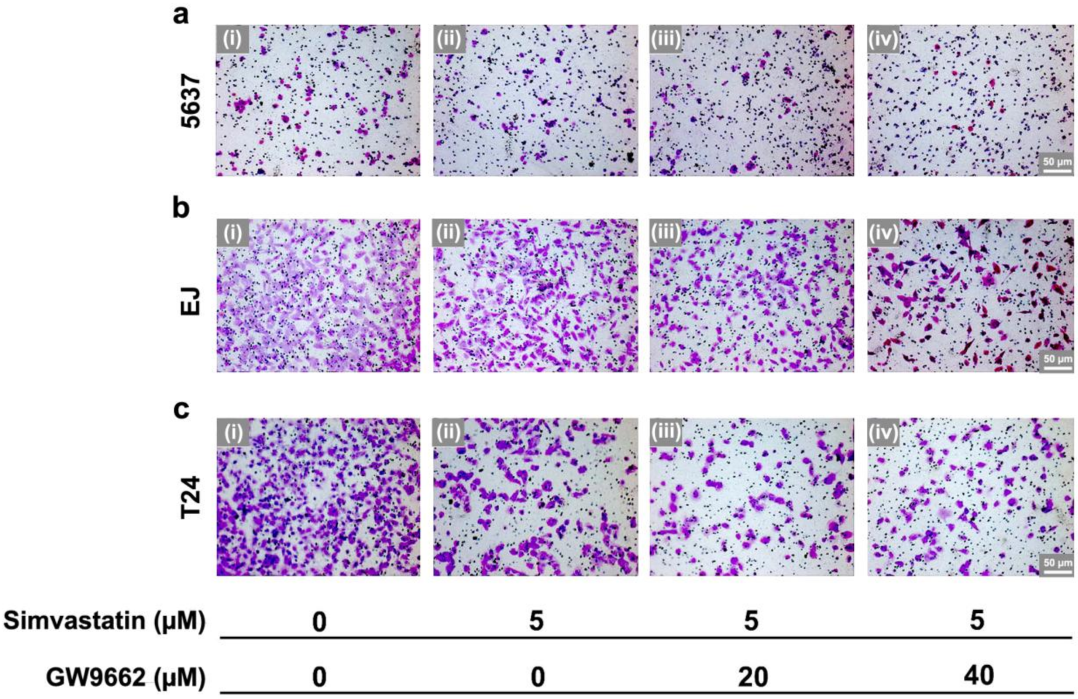

Supplementary Information 2

Supplementary Table S1. Information of the patients and donors.

	**Patients**	**Donors**
**Number**	3	3
**Age, years (Mean ± SD)**	62 ± 1.581	37 ± 2.327
**Gender**	Male	Male
**BCa stage**	Stage II	—
**Surgical method**	Radical resection	—

Note: Stage II means BCa goes into muscle layer of the bladder.

Supplementary Table S2. Significantly altered genes and pathways related with bladder cancer by mRNA microarray using bladder cancer tissues versus normal bladder tissues.

Pathway ID	Pathway Name	Diff Gene Counts in Pathway	Gene Amount in Pathway	Enrichment Score	p-value	FDR	Gene Symbols
4110	Cell cycle	18	124	4.92504304	5.39E-08	3.68E-07	|PTTG1|PLK1|CCNB2|CCNA2|GADD45B|CCNE2|CDC25C|MAD2L1|CDC20|CDK1|CCND1|BUB1|CHEK1|CCND2|CDC7|TTK|CCNB1|CDC6|
4115	p53 signalling pathway	13	68	6.48624949	1.66E-07	1.02E-06	|RRM2|CCNE2|CCNB1|CCND1|THBS1|IGF1|CCND2|CCNG1|CHEK1|CCNB2|GADD45B|CDK1|SERPINE1|
5200	Pathways in cancer	27	327	2.80140063	3.72E-06	1.82E-05	|RUNX1T1|AR|CSF1R|COL4A5|EGLN3|FGF10|NRAS|HHIP|COL4A1|CCND1|IL6|IGF1|KIT|AKT3|RARB|PRKCB|BRCA2|IL8|PTCH1|MMP9|CKS2|MMP1|GLI3|KITLG|HIF1A|FZD7|CCNE2|
3320	PPAR signalling pathway	11	71	5.25646221	1.42E-05	5.46E-05	|ADIPOQ|OLR1|GK|ILK|SCD|PLTP|ACSL1|SORBS1|MMP1|LPL|CD36|
561	Glycerolipid metabolism	9	55	5.55186669	6.13E-05	0.0002154	|LPL|GLA|GK|DGKB|PPAP2B|ALDH1B1|AKR1B10|ALDH3A2|DGKE|

71	Fatty acid degradation	8	44	6.16874077	7.69E-05	0.0002552	|ADH1C|ACAT1|ACADSB|ADH1B|ALDH1B1|ACSL1|ALDH3A2|ADH5|
5219	Bladder cancer	7	38	6.24990841	0.0002156	0.0006606	|NRAS|THBS1|MMP1|MMP9|CCND1|IL8|RPS6KA5|
4012	ErbB signalling pathway	9	88	3.46991668	0.0023192	0.0055989	|CAMK2G|NRAS|AREG|GAB1|EREG|PAK6|AKT3|BTC|PRKCB|
61	Fatty acid biosynthesis	2	6	11.3093581	0.0240649	0.0429218	|ACACB|ACACA|

Supplementary Table S3. List of primers for semiquanitative RT-PCR and qRT-PCR.

Gene	Symbol	Forward primer (5'-3')	Reverse primer (5'-3')	Annealing Temperature (℃)	Length (bp)
Amphiregulin	*AREG*	5'-TGCTGGATTGGAC CTCAATG-3'	5'-TCCCGAGGACGGT TCACTAC-3'	56	163
Epidermal growth factor receptor	*ERBB1*	5'-GGTGCGAATGACA GTAGCATTATGA-3'	5'-AAAGGTGGGCTCC TAACTAGCTGAA-3'	56	184
Erb-b2 receptor tyrosine kinase 2	*ERBB2*	5'-CAGGCACCGCAGC TCATCTA-3'	5'-TCCCAGGTCACCA TCAAATACATC-3'	56	139
Erb-b2 receptor tyrosine kinase 3	*ERBB3*	5'-CCCTGCCATGAGA ACTGCAC-3'	5'-TCACTGTCAAAGC CATTGTCAGAT-3'	56	112
Erb-b2 receptor tyrosine kinase 4	*ERBB4*	5'-TGATAGGCCGTTG GTTGTCTGA-3'	5'-CCAGGTAGACATA CCCAATCCAGTG-3'	56	149
Epiregulin	*EREG*	5'-CTGCCTGGGTTTCC ATCTTCT-3'	5'-GCCATTCATGTCA GAGCTACACT-3'	56	163
GRB2 associated binding protein 1	*GAB1*	5'-ATCAGAAACGCCA GCGAAGA-3'	5'-TCAGATACCACAA AGCACCA-3'	56	209
Glyceraldehyde-3-phosphate dehydrogenase	*GAPDH*	5'-TGCACCACCAACT GCTTAG -3'	5'-GATGCAGGGATGA TGTTC -3'	56	176
Peroxisome proliferator activated receptor alpha	*PPARα*	5'-ACTCTGCCCCCTCT CGCCACTC-3'	5'-GCCAAAGCTTCCA GAACTATCCTC-3'	60	130
Peroxisome proliferator activated receptor delta	*PPARβ*	5'-GAGCAGCCACAGG AGGAAGCC-3'	5'-GCTGTGGTCCCCC AT-3'	56	100
Peroxisome proliferator activated receptor gamma	*PPARγ*	5'-AGAGATGCCATTCT GGCCCAC-3'	5'-GTGGAGTAGAAAT GCTGGAGA-3'	56	128

Supplementary Table S4. List of primary antibodies.

Antigens	Species antibodies raised in	Dilution (IF)	Dilution (WB)	Supplier
E-Cadherin, human	Rabbit, monoclonal	1:200	1:1,000	Cell Signaling Technology, USA, Cat. No: #3195
N-Cadherin, human	Rabbit, monoclonal	1:200	1:1,000	Cell Signaling Technology, USA, Cat. No: #13116
Vimentin, human	Rabbit, monoclonal	1:200	1:10,00	Cell Signaling Technology, USA, Cat. No: #5741
β-Catenin, human	Rabbit, monoclonal	-	1:1,000	Cell Signaling Technology, USA, Cat. No: #8480
Claudin-1, human	Rabbit, monoclonal	-	1:1,000	Cell Signaling Technology, USA, Cat. No: #13255
MMP-2, human	Rabbit, monoclonal	-	1:500	Cell Signaling Technology, USA, Cat. No: #13132
Glyceraldehyde 3-phosphate dehydrogenase (GAPDH), human	Mouse, monoclonal	-	1:2,000	Santa Cruz Biotechnology Inc., USA, Cat. No: sc-365062
Cyclin D1, human	Rabbit, monoclonal	-	1:1,000	Cell Signaling Technology, USA, Cat. No: #2978S
CDK4, human	Rabbit, monoclonal	-	1:1,000	Abcam, UK, Cat. No: ab108357
CDK6, human	Rabbit, monoclonal	-	1:1,000	Abcam, UK, Cat. No: ab124821
Caspase 3, human	Rabbit, monoclonal	-	1:1,000	Cell Signaling Technology, USA, Cat. No: #9665P
Caspase 9, human	Rabbit, monoclonal	-	1:1,000	Cell Signaling Technology, USA, Cat. No: #9508P
Cleaved Caspase 3, human	Rabbit, monoclonal	-	1:1,000	Cell Signaling Technology, USA, Cat. No: #9664P
Cleaved Caspase 9, human	Rabbit, monoclonal	-	1:1,000	Cell Signaling Technology, USA, Cat. No: #7237P
ERBB1, human	Rabbit, monoclonal	-	1:1,000	Abcam, UK, Cat. No: ab52894
PPARγ, human	Rabbit, monoclonal	-	1:1,000	Abcam, UK, Cat. No: ab45036
p-p38, human	Rabbit, monoclonal	-	1:1,000	Cell Signaling Technology, USA, Cat. No: #9211S
t-p38, human	Rabbit, monoclonal	-	1:1,000	Cell Signaling Technology, USA, Cat. No: #9212S
p-AKT, human	Rabbit, monoclonal	-	1:1,000	Cell Signaling Technology, USA, Cat. No: #4060P
t-AKT, human	Rabbit, monoclonal	-	1:1,000	Cell Signaling Technology, USA, Cat.

				No: #4691L
p-GSK3β, human	Rabbit, monoclonal	-	1:1,0000	Cell Signaling Technology, USA, Cat. No: #5558S
t-GSK3β, human	Rabbit, monoclonal	-	1:1,0000	Cell Signaling Technology, USA, Cat. No: #12456S

Supplementary Table S5. List of secondary antibodies and counterstaining of nuclei.

Secondary detection system used	Host	Method	Dilution	Supplier
Anti-Mouse-IgG (H+L)-HRP	Goat	WB	1:10,000	Sungene Biotech, China, Cat. #LK2003
Anti-Rabbit-IgG (H+L)-HRP	Goat	WB	1:5,000	Sungene Biotech, China, Cat. #LK2001
Anti-rabbit IgG (H+L), F (ab') 2 Fragment (Alexa Fluor 555 Conjugate)	Goat	IF	1:50	Cell Signaling Technology, USA, Cat. #4413
Hoechst 33342 (1 mg/ml) nucleic acid staining (DAPI)	-	IF	1:750	Molecular Probes/Invitrogen, Carlsbad, CA, USA, Cat. #A11007
